# Forecasting invasive mosquito abundance in the Basque Country, Spain using machine learning techniques

**DOI:** 10.1186/s13071-025-06733-y

**Published:** 2025-03-15

**Authors:** Vanessa Steindorf, Hamna Mariyam K. B., Nico Stollenwerk, Aitor Cevidanes, Jesús F. Barandika, Patricia Vazquez, Ana L. García-Pérez, Maíra Aguiar

**Affiliations:** 1 M3A, Basque Center for Applied Mathematics, Mazarredo 14, 48009 Bilbao, Bizkaia Spain; 2 Ikerbasque, Basque Foundation for Science, 48009 Bilbao, Bizkaia Spain; 3 Animal Health Department, NEIKER-Basque Institute for Agricultural Research and Development, Basque Research and Technology Alliance (BRTA), 48160 Derio, Bizkaia Spain

**Keywords:** Mosquito eggs, Dengue, *Aedes albopictus*, Machine learning, Vector-borne diseases, Entomological surveillance

## Abstract

**Background:**

Mosquito-borne diseases cause millions of deaths each year and are increasingly spreading from tropical and subtropical regions into temperate zones, posing significant public health risks. In the Basque Country region of Spain, changing climatic conditions have driven the spread of invasive mosquitoes, increasing the potential for local transmission of diseases such as dengue, Zika, and chikungunya. The establishment of mosquito species in new areas, coupled with rising mosquito populations and viremic imported cases, presents challenges for public health systems in non-endemic regions.

**Methods:**

This study uses models that capture the complexities of the mosquito life cycle, driven by interactions with weather variables, including temperature, precipitation, and humidity. Leveraging machine learning techniques, we aimed to forecast *Aedes* invasive mosquito abundance in the provinces of the Basque Country, using egg count as a proxy and weather features as key independent variables. A Spearman correlation was used to assess relationships between climate variables and mosquito egg counts, as well as their lagged time series versions. Forecasting models, including random forest (RF) and seasonal autoregressive integrated moving average (SARIMAX), were evaluated using root mean squared error (RMSE) and mean absolute error (MAE) metrics.

**Results:**

Statistical analysis revealed significant impacts of temperature, precipitation, and humidity on mosquito egg abundance. The random forest (RF) model demonstrated the highest forecasting accuracy, followed by the SARIMAX model. Incorporating lagged climate variables and ovitrap egg counts into the models improved predictions, enabling more accurate forecasts of *Aedes* invasive mosquito abundance.

**Conclusions:**

The findings emphasize the importance of integrating climate-driven forecasting tools to predict the abundance of mosquitoes where data are available. Furthermore, this study highlights the critical need for ongoing entomological surveillance to enhance mosquito spread forecasting and contribute to the development and assessment of effective vector control strategies in regions of mosquito expansion.

**Graphical Abstract:**

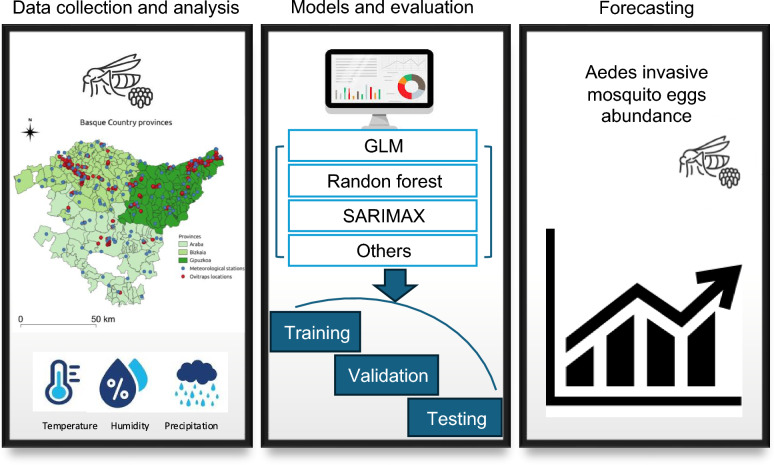

**Supplementary Information:**

The online version contains supplementary material available at 10.1186/s13071-025-06733-y.

## Background

Vector-borne diseases, particularly those transmitted by mosquitoes, have become a significant global concern. The expansion of mosquitoes and the increase in transmitted diseases are escalating worldwide [1]. In the Americas, dengue cases alone surpassed 7 million by May 2024, exceeding the total annual of 4.6 million cases reported in the previous year [2]. Traditionally, these diseases primarily affected tropical and subtropical regions [1–3]. However, climate change and global warming are facilitating the spread, adaptation, and establishment of competent mosquitoes into temperate zones previously unaffected by such diseases, such as Europe [4]. In addition, increased human mobility also plays a critical role, as travelers returning from endemic areas to nonendemic regions may introduce infections (imported cases), potentially sparking local transmission in areas with competent vectors and susceptible populations. Recently, countries such as France, Italy, and Spain have experienced a significant rise in dengue imported cases. In France, from the beginning of 2024 up to June of the same year, the imported cases overpasses the 200 cases recorded over the whole previously year (in 2023) [5]. Moreover, around 500 imported cases were registered in Italy. In addition, there has been a marked increase in autochthonous cases, with 85 reported in France and 207 in Italy [1].

In the Basque Country, an autonomous community in northern Spain, no autochthonous cases of *Aedes* mosquito-borne diseases have been recorded to date. However, with the lifting of mobility restrictions after the severe acute respiratory syndrome coronavirus 2 (SARS-CoV-2) pandemic, the Public Health Epidemiological Unit in the Basque Country has registered an increase of dengue, chikungunya, and Zika imported cases [6]. On the other hand, entomological surveillance in various localities has shown an increase in the abundance of *Aedes albopictus* eggs, and the establishment of *Aedes japonicus* populations [7]. These developments highlight the critical importance of maintaining robust surveillance systems, as effective monitoring is essential for preventing and controlling the spread of arboviruses.

The mosquitoes undergo to three life stages before becoming adults: egg, larva, and pupa. Female mosquitoes search for human blood since it provides the essential nutrients required for egg development. After feeding, the female typically rests while her eggs mature and then lays them in small batches in areas with stagnant water, such as containers, tire ruts, or tree holes. A female mosquito can lay an average of 200–400 eggs at a time [8, 9]. Most eggs hatch into larvae within 48 h if still water is available. However, they can survive several days, from 300 to 400 days, without coming into contact with water [8, 9]. This reproductive process is strongly influenced by environmental factors such as temperature, humidity, and rainfall, which affect the availability of suitable breeding sites and ultimately the success of egg development.

The worst conditions for *A. albopictus* eggs are high temperatures and low relative humidity [10]. Egg mortality decreases with increasing relative humidity and median temperatures of 24–26 °C. Conversely, the optimum temperature for females to lay eggs is between 25 °C and 30 °C. At temperatures of 20 °C and 34 °C, mosquitoes lay significantly fewer eggs [10, 11]. The optimal temperature for the development and survival of *A. albopictus* occurs at summer temperatures of 25–30 °C. While a mean winter temperature of more than 0 °C allows egg survival, a mean annual temperature of more than 11 °C is required for adult activity [1]. At least 500 mm of annual rainfall is required for the breeding habitat, although mosquito populations have been established in areas with lower rainfall [12]. In contrast, periods of high precipitation temporarily reduce the number of females actively searching for a host. The reproductive season is influenced by increasing temperatures in spring and the onset of egg diapause in autumn, triggered by daylight hours below 13–14 h [1, 12].

The association between climate factors and the prediction of dengue outbreaks has been widely studied [13–19]. By employing machine learning approaches, particularly those applied in endemic regions, have shown promise in enhancing the accuracy of dengue outbreak forecasts. On the other hand, some studies have incorporated vector data, such as adult mosquito populations, as proxies [16, 20–22], or larvae abundance [23]. Moreover, the role of *Aedes aegypti* abundance, climatic factors, and disease surveillance has been also evaluated in regions where autochthonous dengue transmission was recently introduced, such as in southern Brazil [16].

However, one of the key challenges in fitting and validating predictive models is the necessity of local incidence data on mosquito-borne diseases cases and vector surveillance information. These data serve as a critical predictor variable for outbreak forecasting, but it is typically only available in endemic regions, where autocthonous cases is a persistent public health concern. Unfortunately, such data are often limited or spatially restricted due to various factors, primarily the high costs associated with collecting and maintaining accurate, up-to-date surveillance systems, making it difficult to obtain comprehensive data for nonendemic or under-resourced regions [24].

Despite these challenges, numerous studies have successfully used climate variables and also historical data on mosquito adult abundance as proxies to forecast mosquito abundance. For example, mosquito abundance has been predicted using artificial neural network (ANN) models [21, 25], with some studies using adult mosquito populations as predictors [21], while others employed mechanistic models [26]. Another study used an ordinary differential equation (ODE) model to predict mosquito abundance, considering temperature, rainfall, egg diapause, and population dynamics of mosquitoes in southern France [27]. Nonetheless, this study did not include humidity as a climate factor, and prior hypotheses based on vector-related parameters were necessary, drawn from existing literature.

Finally, only a few studies have considered mosquito eggs as predictors for temporal forecasting [13, 15, 28]. A more recent study employed spatio-temporal forecasting using stacked machine learning techniques [29]. Most studies that have used egg counts for forecasting have linked them with climate changes and ovitrap data to predict dengue outbreaks in endemic regions. However, in nonendemic areas such as the Basque Country, where there is no local *Aedes* mosquito-borne diseases transmission and adult mosquito populations are not systematically monitored, predicting mosquito abundance becomes crucial for controlling the spread of the disease and informing surveillance and intervention strategies.

In this study, we aim to estimate *Aedes* invasive mosquito abundance in a region where autochthonous mosquito-borne diseases transmitted by *A. albopictus* (such as dengue) have not yet been recorded, for example, the Basque Country. By using the available data from the Basque Country’s provinces, we use machine learning techniques to model the relationship between recorded mosquito ovitrap egg counts and key environmental factors, including temperature, humidity, and precipitation. In the “Materials and methods” section, the relationship between climate variables and the abundance of mosquito eggs is analyzed within the context of a maritime climate, as the Basque Country, at the provincial and municipality levels. We explore and compare different machine learning models, considering variations such as including and excluding lagged versions of egg counts as a predictor, in the “Results” section. Notably, incorporating lagged versions of both independent and dependent variables consistently improves the performance of most models, demonstrating the importance of temporal dependencies in mosquito abundance forecasting. Further, in the “Results” section, fitting the best-performing models to the available data on recorded egg counts in ovitraps allow us to produce more accurate predictions of invasive mosquito abundance.

## Methods

### Entomological and meteorological data

**Fig. 1:**
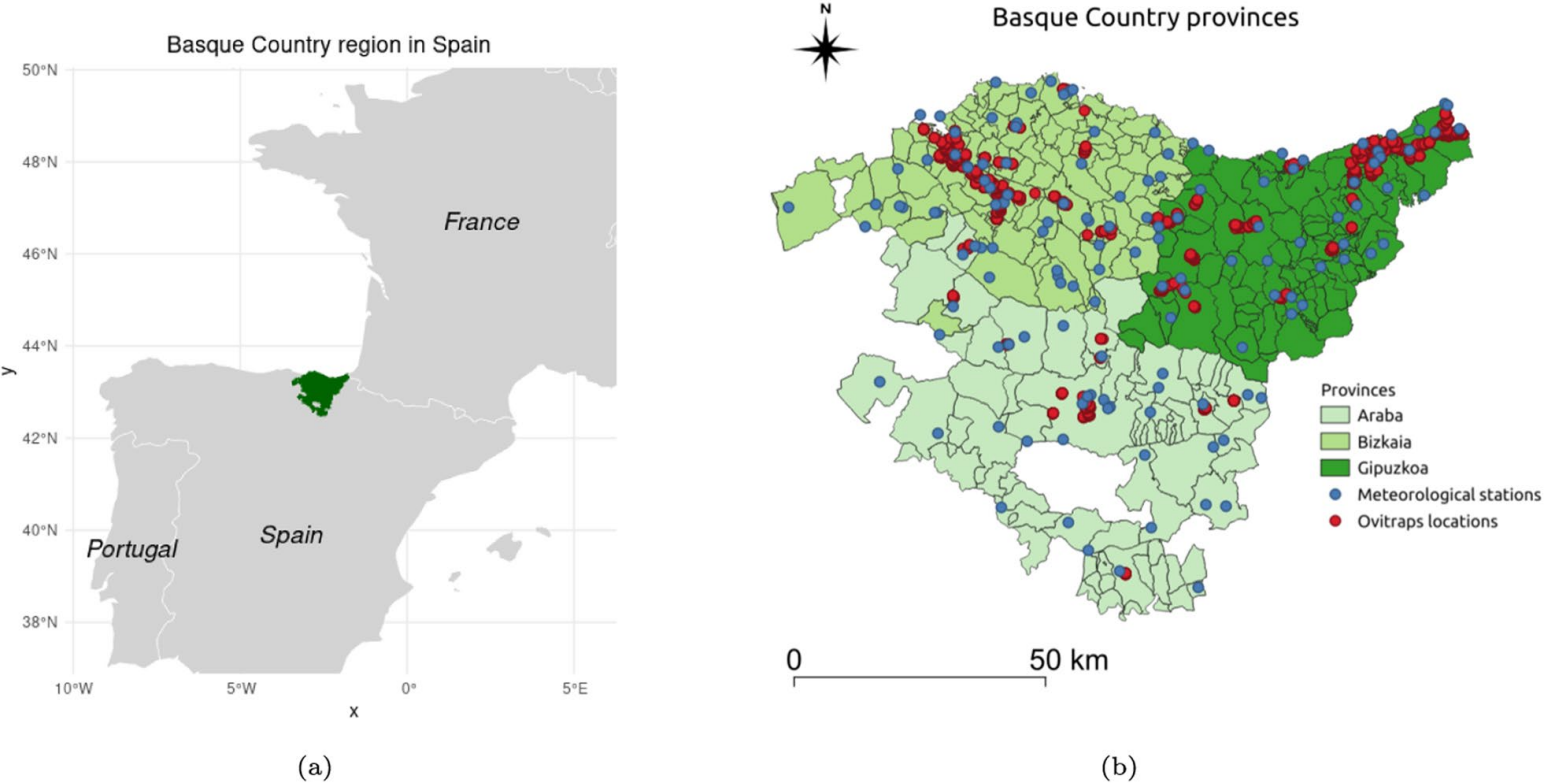
**a** Basque Country region in Spain (location in the European map). **b** Meteorological stations and ovitraps locations in the Basque Country provinces during the intersection study period (2016–2023)

Data on *Aedes* mosquito egg counts from 2013 to 2023 in the Basque Country were obtained using ovitraps as described in [7, 30]. Following the European Centre for Disease Prevention and Control (ECDC) recommended guidelines [1], the ovitraps were distributed across the three provinces, covering 63 municipalities, as shown in Fig. 1b.

The number of ovitraps varies by municipality, with two sampling areas selected in most cases. Each sampling area typically contains five ovitraps, which are positioned in sheltered spots away from direct sunlight and wind, often hidden within vegetation. Therefore, up to ten ovitraps per municipality were placed in most cases. Each ovitrap contains water and a wooden stick (or tablex) that serves as a substrate for mosquito egg-laying. Every 14 days (on average), these paddles are removed, and new ones are put in their place. Thus, each municipality and area is sampled roughly 10–12 times per year, from June through November [7].

Meteorological data for the Basque Country were collected from the Basque Meteorological Agency (Euskalmet) across several weather stations (see Fig. 1b),^1^ covering the period from 2016 to 2023. The data, obtained from the OpenData Euskadi website [31], include precipitation [recorded as cumulative precipitation in millimeters (mm) or liters per square meter (l/m^2^)], temperature [measured in degrees Celsius (°C)], and humidity [relative air humidity as a percentage (%)]. Weather observations were recorded every 10 min at each station. For this study, we calculated daily averages of temperature and humidity and daily cumulative precipitation for each meteorological station.

#### Study area and data per provinces

The Basque Country, located in northern Spain, is divided into three administrative provinces: Araba (Álava), Bizkaia (Biscay), and Gipuzkoa (see Fig. 1). With a total area of 7234 km^2^ and a population of approximately 2.18 million [32], the region is characterized by diverse landscapes and a maritime climate, with temperate conditions and high annual precipitation, particularly in the coastal areas. Araba, the southernmost province, has a more continental influence in its climate, with drier and slightly colder conditions than the coastal provinces of Bizkaia and Gipuzkoa. Bizkaia and Gipuzkoa, bordered by the Cantabrian Sea, experience milder temperatures and higher humidity. These climatic differences across the provinces influence the mosquito abundance patterns, which this study aims to capture and analyze through the environmental data collected.

For this study, we analyzed ovitrap mosquito egg counts collected in various locations across all three provinces. The data were preprocessed by averaging the 20 highest egg counts per province over a 14-day interval, considering that each municipality had a maximum of 10 ovitraps. This approach was necessary to address inconsistencies in the number of monitored ovitraps over the studied period and to avoid skewing the results with prevalent zero counts. By selecting the 20 largest egg counts, the data reflect meaningful mosquito activity (in at least two distinct locations), effectively filtering out areas with consistently low or zero activity.

Meteorological data, specifically daily precipitation [cumulative precipitation in millimeters (mm)], air temperature [in degrees Celsius (°C)], and relative humidity [percentage (%)], were obtained by averaging daily values from all available meteorological stations in each province. These features were then aggregated over the previous 14 days to maintain consistent time intervals between the entomological and meteorological datasets. The average annual temperature and accumulated precipitation in each province align with environmental conditions favorable for *A. albopictus* survival, approximately 11.5 °C and 878 mm in Araba, 13.8 °C and 1278 mm in Bizkaia, and 13.4 °C and 1610 mm in Gipuzkoa [7], which are consistent with the survival thresholds discussed in the literature for this species [1, 12].

**Fig. 2:**
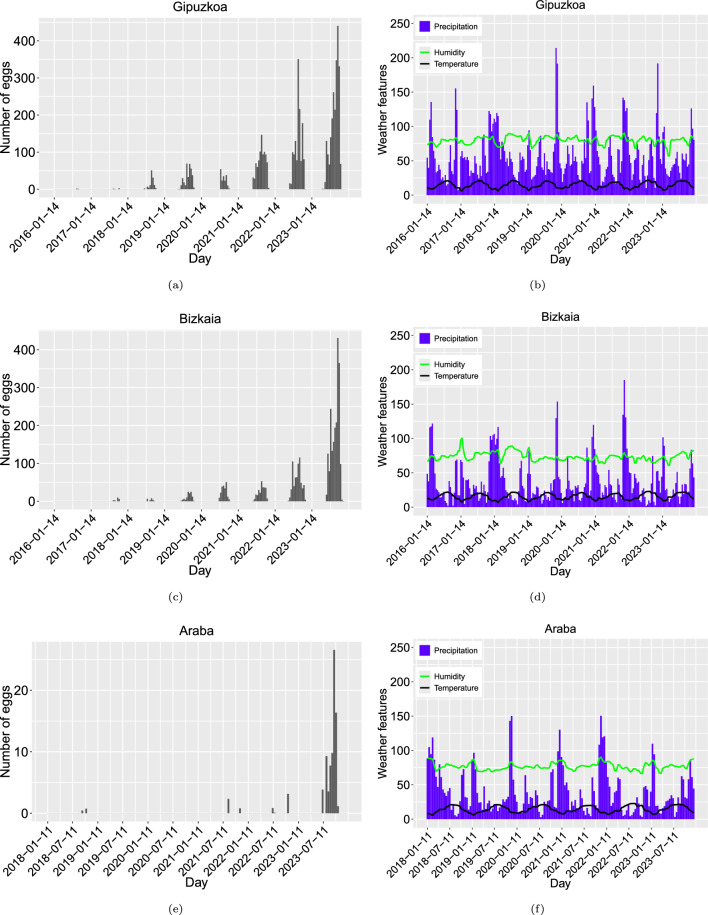
Number of mosquitoes eggs collected in **a**, **c**, **e** and average temperature (°C), relative air humidity (%), and cumulative precipitation (mm) in **b**, **d**, **f**. Data were gathered biweekly for Gipuzkoa, Bizkaia, and Araba, respectively

The time series of the average egg counts, temperature, humidity, and cumulative precipitation for each province in the Basque Country are shown in Fig. 2.

Mosquito eggs are typically found during the summer months, from June to October, when the combination of higher temperatures and favorable humidity conditions promotes their activity and reproduction. As shown in Fig. 2a, the egg count in the entire Gipuzkoa province has significantly increased over the last years of collected data, although this trend may vary between municipalities. For example, in the city of Irun (Supplementary Material S3), the second most populated city in Gipuzkoa, located on the border with France, where variability is present without a clear increasing trend.

In Gipuzkoa, temperature exhibited a clear seasonal annual pattern, while accumulated rainfall showed no apparent trend. Humidity, however, decreased during the winter and followed a quasi-periodic structure (see Fig. 2b). The winter of 2019, right after the expected period of higher egg presence, was exceptionally rainy compared with other winters in the province. Combined with low humidity (below 75%), this may have contributed to the lower egg counts observed in the following summer season (2020). In contrast, the dry summer of 2022, accompanied by higher humidity levels (above 75%), may explain the increased egg counts observed that year.

In Bizkaia, the time series of egg counts has displayed a consistent upward trend over the years, with positive egg traps first recorded in 2017 (Fig. 2c). Notably, the average mosquito egg count in Bizkaia during 2023 serves as a good proxy for the province-wide average, as shown by the time series for Bilbao, the capital of Bizkaia (Fig. S4c in Supplementary Material S3).

The temperature in Bizkaia followed a clear seasonal pattern, while accumulated rainfall showed no apparent trend, with significant cumulative precipitation occurring later in 2021. In contrast to Gipuzkoa, however, humidity in Bizkaia exhibited periodic increases approximately every 2 years, with higher levels typically observed during winter months (Fig. 2d).

Moreover, average precipitation in Bizkaia was slightly lower than in Gipuzkoa. Temperature fluctuations in Bizkaia were more pronounced, as indicated by the steeper slope of its temperature curve compared with Gipuzkoa, potentially explaining the lower average egg counts in the region. In addition, the dry summer of 2021, followed by a rainy winter, may have contributed to the consistent egg count trend observed.

Furthermore, although ovitraps have been distributed and data collected in the province of Araba since 2013, positive egg traps were not recorded until 2018, with no positive ovitraps observed in 2019 or 2020 (Fig. 2e). In Laudio, the second most populated municipality in Araba, positive ovitraps were only recorded in 2021 (Fig. S4e in Supplementary Material S3).

The average temperature in Araba exhibits annual seasonality, while precipitation lacks a clear trend, though cumulative rainfall is typically higher during winter. On the other hand, humidity also tends to increase alongside precipitation (Fig. 2f). The lower average temperature in this province may contribute to the reduced presence of mosquito eggs.

Given the dispersed nature of data in Araba, with many zero values in egg counts (Fig. 2e), there is insufficient information to develop a reliable training dataset for model fitting. Therefore, this province is excluded from further analysis. Smaller spatial units, such as individual municipalities, are similarly excluded, with the focus of this study being the two Basque Country provinces, Gipuzkoa and Bizkaia. Nonetheless, descriptive statistics and detailed analyses at the municipal level for Irun and Bilbao, which have adequate data, are provided in Supplementary Material S3.1 and S3.2, respectively.

### Methodological approach

#### Data processing

After gathering data, preprocessing is a crucial initial step before model training, forecasting, and evaluation. In this study, data preprocessing included the following steps. First, we ensured a consistent interval for both the independent and dependent variables, selecting a biweekly interval for the entomological data based on the average 14-day period in which egg counts were collected.

Next, we addressed missing values through imputation, filling gaps with zero values. This choice is scientifically justified within the context of this dataset, as institutional data indicated that, for months without data collection, ovitrap counts would have likely been zero [7]. This assumption was based on data from four sentinel points (two in Gipuzkoa and two in Bizkaia) monitored over a year to determine the start and end of *Aedes* mosquito activity in regions with recorded presence in the previous year.

Moreover, we included only the 20 highest egg counts at the provincial level to account for variations in the number of monitored ovitraps over time, helping to reduce dataset skewness. Outliers were then removed using a central moving average as a smoothing method, commonly applied to mitigate white noise, random fluctuations, and extreme values [33].

For the meteorological data, no imputation was required as daily weather data were available for the entire study period. In this case, outliers were retained as they could signal significant events associated with the presence or absence of mosquito eggs. Basic exploratory analysis was then conducted using descriptive statistics and correlation tests, incorporating both the original and lagged versions of the meteorological data.

Finally, we split the data into training and testing sets, with the training data comprising $85.71\%$ and $83.33\%$ for Gipuzkoa and Bizkaia, respectively. The remaining 26 data points (1 year of biweekly data) were allocated for testing.

#### Models

In this study, we applied different models including and excluding the lagged version of eggs count as a proxy and the lagged version of the independent environmental variables. To appropriately handle the discrete and non-negative nature of counts, we restrict our choices and applications of the models presented here [34–41]. More details about each model can be found in Supplementary Material S1.

We implement the generalized linear model (GLM), seasonal autoregressive integrated moving average with exogenous variables (SARIMAX), random forest (RF), and conditional inference tree (CTree) models (and other models discussed in Supplementary Material S1) in the R computing language (R version 3.6.3) using the packages MASS, forecast, randomForest and party, respectively. Nevertheless, only the four models cited earlier will be presented in this study because (as discussed in Supplementary Material S1) some models exhibit over-fitting, others demonstrate under-fitting (as is the case with the ANNs model), and some fail to capture any significant features of the data.

#### Stationary analysis

We applied the augmented Dickey–Fuller (ADF) test, a commonly used method for testing the presence of a unit root in time series data, to assess whether the time series is nonstationary [17]. Nonstationarity in a time series often presents means, variances, and covariances that change over time, making the series unpredictable and challenging to model or forecast. Although some models, such as SARIMAX, can handle nonstationarity, stationary time series often yield more reliable results [42].

The null hypothesis of the ADF test states that the series contains a unit root, indicating nonstationarity, while the alternative hypothesis suggests that the series is stationary. To test the null hypothesis, we computed the *P* value. A *P* value less than 0.05 leads us to reject the null hypothesis, implying stationarity.

We conducted the ADF test using the tseries package in R. For both datasets, Gipuzkoa and Bizkaia, the ADF test on the predictor variable yielded a *P* value of approximately $P< 0.01 < 0.05$, indicating that the datasets are stationary.

#### Evaluation metrics

To compare the performance of statistical and machine learning models, three widely used evaluation metrics were employed: the mean absolute error (MAE), the root mean squared error (RMSE), and the *R-squared* ($R^2$) score.

The MAE is calculated as:

1 $$\begin{aligned} {\text{MAE}} = \frac{1}{n} \sum _{i=1}^{n} |y_i - \hat{y}_i|, \end{aligned}$$

where $y_i$ and $\hat{y}_i$ represent the observed and predicted values, respectively, and $| \cdot |$ denotes the absolute value [43]. MAE measures the average magnitude of the errors in a set of predictions, without considering their direction.

The RMSE is given by:

2 $$\begin{aligned} {\text{RMSE}} = \sqrt{\frac{1}{n} \sum _{i=1}^{n} (y_i - \hat{y}_i)^2}, \end{aligned}$$

where $y_i$ and $\hat{y}_i$ are the observed and predicted values, respectively. RMSE gives a higher weight to large errors compared with MAE and is sensitive to outliers.

The $R^2$ score, also known as the coefficient of determination, is calculated as:

3 $$\begin{aligned} R^2 = 1 - \frac{S_r}{S_t} = 1 - \frac{\sum _{i=1}^{n} (y_i - \hat{y}_i)^2}{\sum _{i=1}^{n} (y_i - \overline{y})^2}, \end{aligned}$$

where $S_r$ is the residual sum of squares, representing the sum of squared differences between the observed values ($y_i$) and the predicted values ($\hat{y}_i$); and $S_t$ is the total sum of squares, calculated as the sum of squared differences between the observed values ($y_i$) and their mean ($\overline{y}$). An $R^2$ score of 1 indicates that the model explains all the variability of the response variable, while a score of 0 indicates no explanatory power.

The selection of the best model is based on achieving the lowest MAE or RMSE values or an $R^2$ score closest to 1. In this study, the MAE is chosen as the primary evaluation metric due to its suitability for machine learning models [43].

## Results

### Exploratory statistical analysis

Basic exploratory statistical analysis was performed, starting with descriptive statistics for both the response and predictor variables (after preprocessing and smoothing). All variables in the dataset were found to be skewed and over dispersed. The null hypothesis of normal distribution was rejected based on the results of the Kolmogorov–Smirnov test and the Shapiro–Wilk test, both of which yielded *P* values $P < 0.05$, indicating significant deviation from normality for all variables.

For the Gipuzkoa dataset:

- Eggs count had a mean of 25 and a median of 0.
- Temperature had a mean of 14.6 °C and a median of 14.2 °C.
- Relative air humidity had a mean of 79.9% and a median of 80.8%.
- Precipitation had a mean of 57.6 mm and a median of 49.4 mm.

For the Bizkaia dataset:

- Eggs count had a mean of 16 and a median of 0.
- Temperature had a mean of 15.5 °C and a median of 14.8 °C.
- Relative air humidity had a mean of 73.6% and a median of 72.8%.
- Precipitation had a mean of 37.4 mm and a median of 27.1 mm.

Additional details are provided in Supplementary Material S2.1 (Fig. S1), as well for the province of Araba.

**Fig. 3:**
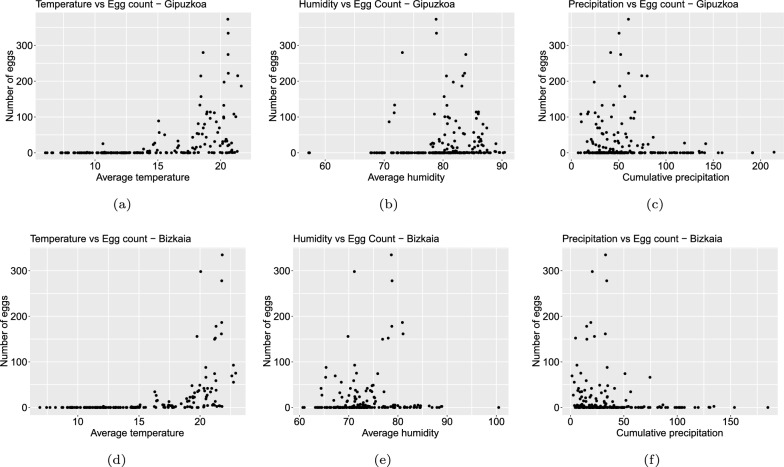
Average temperature (in °C) versus the number of collected mosquito eggs, in **a**, **d**. Average relative air humidity (in %) versus the number of collected mosquito eggs, in **b**, **e**. Accumulated precipitation (in mm) versus the number of collected mosquito eggs, in **c**, **f**. Data gather biweekly, in Gipuzkoa and Bizkaia, respectively

The relationship between meteorological variables and the number of mosquito eggs was explored using scatter plots (Fig. 3a–c for Gipuzkoa and 3d–f for Bizkaia). No linear relationship was confirmed, as indicated by Pearson’s correlation index. Nevertheless, it is well known that the combination of high temperatures (22–27 °C) and high humidity increases oviposition rates (egg laying) in adult female mosquitoes [10, 11]. This association is reflected in Fig. 3a and b for Gipuzkoa, and Fig. 3d and e for Bizkaia.

As described in the “Data processing” section, the time series data were smoothed using a central moving average to reduce short-term fluctuations and noise. This preprocessing step helped mitigate spurious short-term correlations and revealed underlying long-term relationships between variables, thereby increasing correlation indexes.

**Fig. 4:**
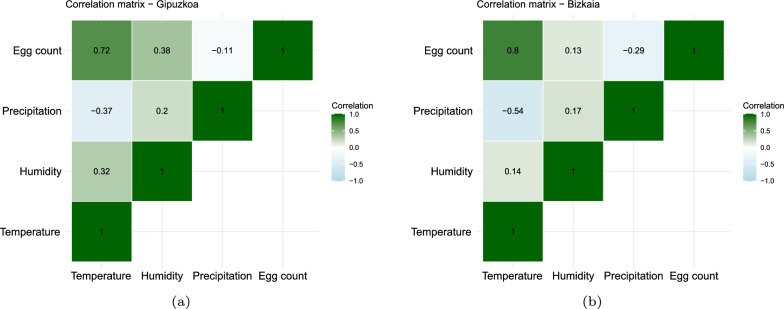
Spearman correlation matrix between weather features and the number of mosquito eggs. The matrix shows a high correlation between the number of eggs and temperature (index ${\text{rs}} = 0.72$ for Gipuzkoa, in **a** and index ${\text{rs}} = 0.8$ for Bizkaia, in **b**) but no significant correlation with the other features

On the other hand, Spearman’s correlation analysis confirmed a strong monotonic relationship between the number of eggs and temperature, with a correlation index $rs \ge 0.72$ (Fig. 4a for Gipuzkoa and 4b for Bizkaia). Although no significant correlations were found between egg counts and the other climate variables, the direction and strength of these relationships are displayed in Fig. 4a and b. Specifically, as humidity increases, the number of eggs increases, showing an intermediate correlation. In contrast, as accumulated precipitation increases, the number of eggs decreases, albeit with very low or negligible correlation.

**Fig. 5:**
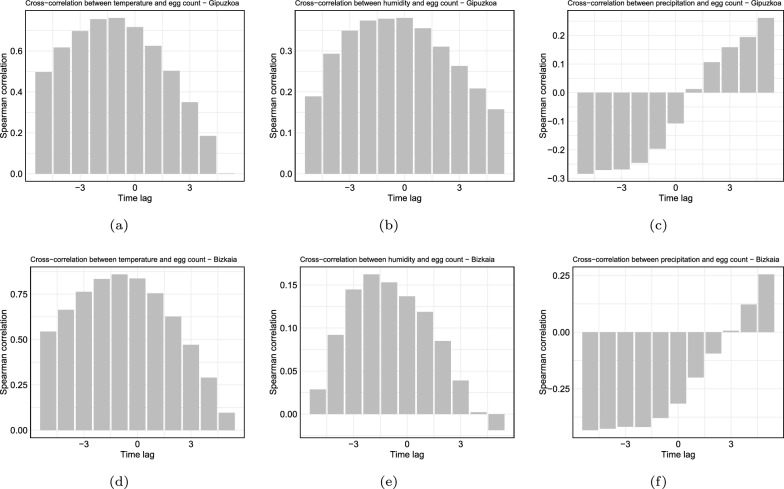
Spearman correlation between the lagged time series of weather features and the number of mosquito eggs, with a time lag of 1 unit (2 weeks). For temperature, the maximum correlation occurs at a lag of $-1$ unit, in **a**, **d**. For humidity, the maximum correlation occurs at a lag of 0 units and $-2$ units, for Gipuzkoa and Bizkaia, in **b**, **e**, respectively. For precipitation, the maximum correlation occurs at a lag of $-5$ units, in **c**, **f**

In addition, we created lagged time series for all the meteorological variables (Fig. 5a–f), and we evaluated the monotonic correlation using Spearman correlation, highlighting the time lag at which the highest index value occurs (Figs. S2a and S3a in Supplementary Material S2). At the time lag at which the highest correlation value occurs, the lagged time series will be used as predictor variables (Figs. S2b and S3b for Gipuzkoa and Bizkaia, in Supplementary Material S2.2 and S2.3, respectively).

For instance, Fig. 5a shows that the highest correlation value between egg counts and temperature series, in Gipuzkoa, occurs at lag −1. This could imply that the egg production series is most strongly correlated with the temperature series 2 weeks (1 period) earlier. Therefore, changes in temperature might have a leading effect on egg production, where temperature changes influence egg production with a delay of 1 period (2 weeks). For humidity (Fig. 5b), the highest correlation occurs at 0 units (0 weeks) with a (low) positive correlation, while for precipitation, (Fig. 5c), the highest correlation occurs at lag −5 units (10 weeks) with a negative (low) correlation.

For Bizkaia, Fig. 5d shows that the highest correlation value between egg counts and temperature series occurs at lag −1. This could imply that the egg production series is most strongly correlated with the temperature series 2 weeks (1 period) earlier. Therefore, changes in temperature might have a leading effect on egg production, where temperature changes influence egg production with a delay of 1 period (2 weeks). For humidity, (Fig. 5e), the highest correlation occurs at lag −2 units (4 weeks) with a positive correlation, while for precipitation, (Fig. 5f), the highest correlation occurs at lag −5 units (10 weeks) with a negative correlation.

High correlation is shown between egg counts and temperature at lag −1, with an index value of 0.76 and 0.83 for Gipuzkoa and Bizkaia, respectively (Figs. S2b and S3b in Supplementary Material S2). While intermediate to low correlation appears to be positive and correlated between humidity and egg counts, Fig. 3b and e show that the highest egg count occurs when humidity percentages are between $70\%$ and $80\%$.

Moreover, a low negative correlation between precipitation and egg counts was found (Fig. 5c and f). Although the strength of the correlation is considered low, the opposite direction in the correlation for precipitation approximately 10 weeks prior to egg collection (almost 3 months earlier) can be explained by the fact that periods of high precipitation temporarily reduce the number of females actively searching for a host and, therefore, laying eggs [1]. On the other hand, drier periods occurring 10 weeks before the collection increase the egg counts. This can be attributed to the fact that mosquito eggs are extremely resistant. They can remain viable in a dry state within a container for 300–400 days without direct water contact, allowing them to stay in ovitraps for extended periods without hatching [9].

### Fitting and error analysis

**Fig. 6:**
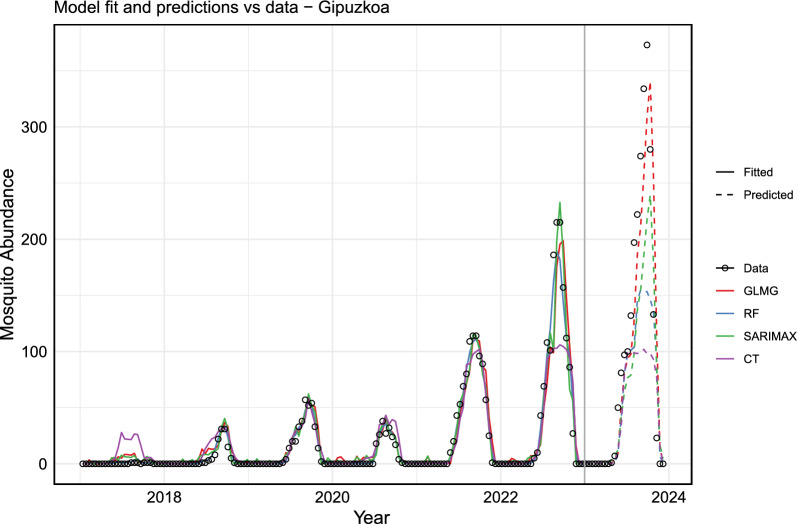
Comparison of actual data with the fitted and test values for Gipuzkoa. The actual data are represented by open black circles, while the fitted values are shown as solid lines and the test values as dashed lines. The models are represented as follows: in blue, the random forest (RF) model (ntree = 600, mtry= 5); in red, the generalized linear model (GLMG); in green, the SARIMAX model; and in purple, the conditional inference trees (CT) model (ntree = 500, mtry = 3). The vertical gray line separates the training dataset (2017–2022) from the test dataset (2023)

**Fig. 7:**
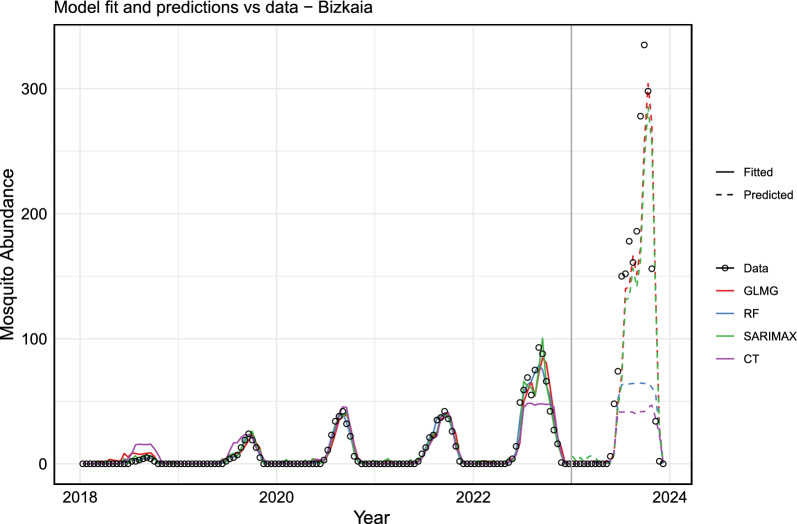
Actual data versus the fitted and test values of the models for Bizkaia. The actual data are represented by open black circles, while the fitted values of each model are shown with solid lines and the test values with dashed lines. In blue, the RF model (ntree = 600, mtry = 5); in red, the GLMG; in green, the SARIMAX model; and in purple, the CT model (ntree = 500, mtry = 3). The vertical gray line delineates the training dataset (2018–2022) from the test dataset (2023)

Prior to model fitting, the dataset was divided into training and testing sets. For Gipuzkoa, the training dataset includes data from 2017 to 2022 ($85.71 \%$), while the test dataset consists of data points from the year 2023 (Fig. 6). In contrast, for Bizkaia, the training dataset covers the period from 2018 to 2022 ($83.33 \%$), with 2023 as the test dataset (Fig. 7). The choice of yearly data is related to the frequency of data availability, while the starting point corresponds to the need for cleaning the missing values (NA, not available) due to the lagged versions of variables.

We train several models on the training dataset, considering, lagged version of the independent variables, as well as including and excluding lagged version of the eggs count variable. The majority of models performed better including the proxy lagged version. Here, we include only models with the best performances, which include the random forest (RF) model, the generalized linear model (GLM) with Gaussian distribution (here abbreviated by GLMG), the seasonal autoregressive integrated moving average with exogenous variables (SARIMAX) model, and the conditional inference trees (CTree) (abbreviated here by CT).

We implement the GLMG, SARIMAX, RF, and CT models in the R computing language (R version 3.6.3) using the glm( ), auto.arima(), randomForest() and cforest() function, respectively. We employed and compare the models on the training dataset and on the testing dataset (Fig. 6). Later, we evaluate each models performance in the datasets using MAE, RMSE, and $R^2$ metrics.

Based on the evaluation metrics, the best performance on the training dataset for Gipuzkua is the RF model. The model could explain $98 \%$ of the variance in the data, according to the $R^2$ evaluation, while in the test dataset $60 \%$. For the test dataset, GLMG was the model that performed better, explaining $85 \%$ of the variance in the dataset, followed by the SARIMAX model (Table 1).

**Table 1:** Error metrics for each chosen model, on the training and test datasets for Gipuzkoa

Model	MAE train	MAE test	RMSE train	RMSE test	$R^{2}$ train	$R^{2}$ test
RF	**2.59**	41.67	**5.73**	73.94	**0.98**	0.60
SARIMAX	4.77	38.73	9.85	61.03	0.94	0.73
GLM	6.09	**29.37**	12.32	**45.71**	0.90	**0.85**
CT	7.62	53.51	17.47	95.49	0.81	0.34

Although the RF model performed best during training, its predictions ranked in third place, which might suggests an over fitting. On the other hand, even though GLMG did not top the training performance, it gave better predictions, making it a more reliable model overall. This suggests that the simplicity of GLMG helped it generalize better to the unseen data, while RF may have captured the noise from the training set, which could reduce the predictive accuracy.

In the case of Bizkaia (Fig. 7), the RF model performed best on the training dataset, explaining $98\%$ of the variance, as indicated by the $R^2$ value. However, on the test dataset, it explained only $15\%$ of the variance. For the test dataset, the GLMG model performed the best, explaining $81\%$ of the variance, closely followed by the SARIMAX model with $80\%$ (Table 2).

**Table 2:** Error metrics for each chosen model, on the training and test datasets for Bizkaia

Model	MAE train	MAE test	RMSE train	RMSE test	$R^{2}$ train	$R^{2}$ test
RF	**1.16**	57.28	**2.84**	97.54	**0.98**	0.15
SARIMAX	2.34	30.23	4.43	47.83	0.95	0.80
GLM	3.31	**27.43**	5.94	**46.43**	0.90	**0.81**
CT	3.97	64.68	8.28	109.37	0.81	$-$0.07

Among the four models evaluated, the CT model performed the worst, based on all error metrics for both the training and test datasets. Moreover, the CT model was unable to explain the test dataset for Bizkaia.

The poor performance of the models on the Bizkaia test set, as shown in the time series, can be attributed to differences in the characteristics of the training and test data. Notably, the mean value of the training data is significantly lower than that of the test data, with egg counts in 2023 being unusually high. This discrepancy between the training and test datasets likely contributed to the models’ suboptimal performance for Bizkaia.

After training, testing, and evaluating each model, we used the models with the best performance to predict future *Aedes* invasive mosquito abundance. For this, we included 2023 data points in the training dataset and, using the historical time series data along with lagged versions of the variables, we forecast values based on the last observations.

**Fig. 8:**
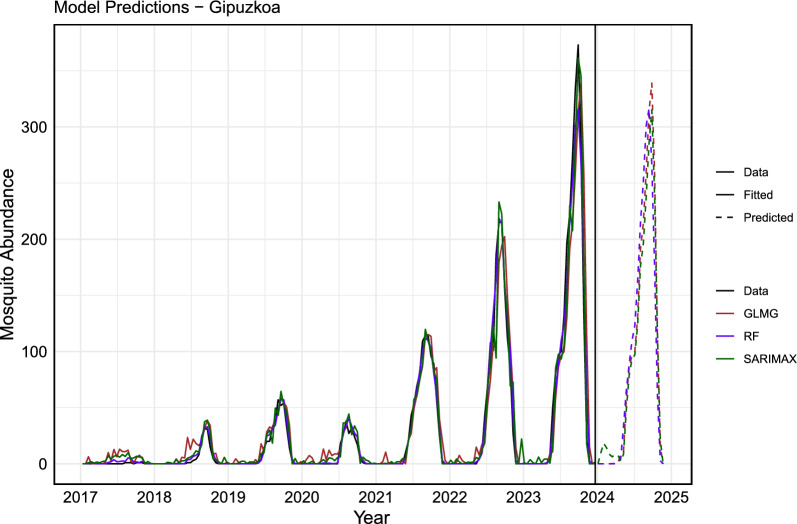
The actual data versus the fitted and predicted values for Gipuzkoa. The actual data are represented by a solid black line. The fitted values for each model are shown as solid colored lines, and the predicted values are displayed as dashed lines. In blue, the RF model (ntree = 600, mtry = 5); in brown, the GLMG model; and in green, the SARIMAX model. The vertical black line delineates the training dataset (from 2017 to 2023) from the forecasted period for the year 2024

**Fig. 9:**
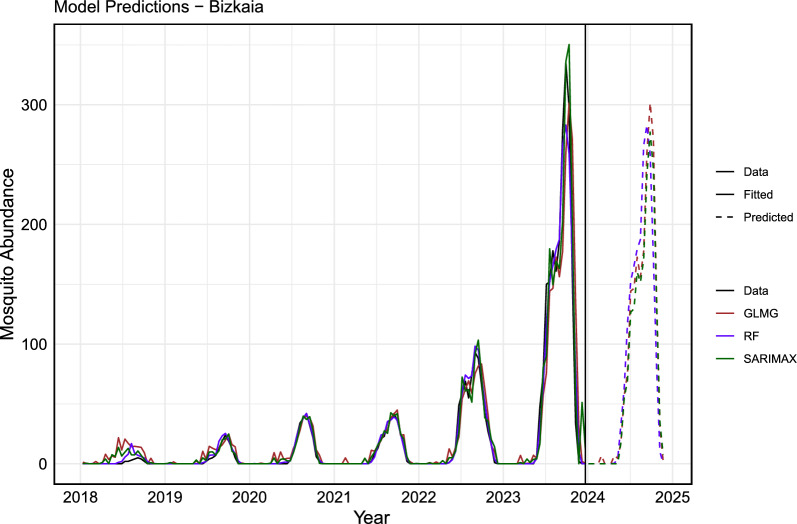
The actual data versus the fitted and predicted values of the model for Bizkaia. The actual data are represented by a solid black line, while the fitted values of each model are shown as solid colored lines and the predicted values as dashed colored lines. In blue, the RF model (ntree= 600, mtry = 5); in brown, the GLMG model; and in green, the SARIMAX model. The vertical black line delineates the training dataset (from 2018 to 2023) from the forecasted period for the year 2024

Figure 8 shows the fitted values and predictions for mosquito abundance in Gipuzkoa for 2024, while Fig. 9 presents the same for Bizkaia. Only the three models with the best performance are displayed. It is noteworthy and expected that extending the training dataset length improved the performance of all models. This highlights the importance of maintaining entomological surveillance for more accurate future predictions.

The error analysis for the training dataset is presented in Tables 3 and 4. The results indicate that, across different metrics, the RF model provided the best fit, explaining $98\%$ of the variance in both Gipuzkoa and Bizkaia, making it suitable for forecasting. The SARIMAX model also performed well, explaining $95\%$ and $94\%$ of the variance in Gipuzkoa and Bizkaia, respectively.

**Table 3:** Error metrics in the training dataset for Gipuzkoa

Model	MAE	RMSE	$R^{2}$
RF	**3.72**	**8.70**	**0.98**
SARIMAX	6.79	14.02	0.95
GLM	9.60	20.06	0.90

**Table 4:** Error metrics in the training dataset for Bizkaia

Model	MAE	RMSE	$R^{2}$
RF	**2.81**	**7.91**	**0.98**
SARIMAX	5.27	12.44	0.94
GLM	7.47	18.82	0.87

At the municipal level (see Supplementary Material S3 for more details), the RF model performed best for Irun (in Gipuzkoa), while for Bilbao (in Bizkaia), the SARIMAX model provided the best fit, explaining $97\%$ of the variance in the training dataset.

Furthermore, we estimate and expect that mosquito abundance in 2024 will be lower compared with the previous year, both at the provincial and municipal levels. This reduction may be due to various factors, such as changes in optimal environmental conditions and potential variations in weather patterns.

## Discussions

The Basque Country, an autonomous community in northern Spain, has experienced an increase in imported cases of mosquito-borne diseases, along with the establishment and expansion of *A. albopictus* and *A. japonicus* mosquitoes. This study uses egg count data retrieved from ovitraps monitored by the regional surveillance program conducted by the Department of Public Health of the Basque Government and the public agency NEIKER at various locations across the Basque provinces. We employ statistical models and machine learning techniques to model the relationship between the recorded mosquito ovitrap egg counts and climate factors such as temperature, humidity, and precipitation.

Before selecting the model, a statistical analysis was conducted on the dataset to examine the influence of environmental factors on the predictor variables. We compared different models, including versions with and without lagged egg counts as a proxy. Importantly, incorporating lagged versions of all independent and dependent variables improved the performance of most models.

We found that forecasting mosquito abundance is particularly challenging in nonendemic areas, where no local mosquito-borne cases have been reported. While environmental factors are the primary drivers of mosquito abundance and distribution, the time series data are not always linearly correlated, which hinder the improvement of forecasting efforts. Nevertheless, temperature shows to be the most important climate feature, while precipitation had less influence. As previously stated, the availability of human water sources appears to have a greater impact on the breeding of invasive *Aedes* mosquitoes than natural rainfall, as these mosquitoes often rely on artificial containers near human habitats [44]. Although heavy rainfall can disrupt larval development by washing out breeding sites, the connection between precipitation and mosquito populations varies depending on local climate conditions [44].

In addition, the inclusion of egg abundance proved to be a key predictor. Our findings confirm that incorporating mosquito-related data improves the fitting and forecasting of predictive models. Consequently, continuous monitoring of mosquitoes and egg abundance by public health systems is essential for more accurate forecasting and effective control measures.

Furthermore, selecting the appropriate lagged variables and ovitrap egg counts, we validated the models using different evaluation metrics. Based on metrics such as root mean squared error (RMSE) and mean absolute error (MAE), the random forest (RF) model outperformed the others, followed by the seasonal autoregressive integrated moving average with exogenous variables (SARIMAX) model. Among the models evaluated, RF performed best on the training data, while the generalized linear model (GLM) performed best on the testing data, with SARIMAX in second place.

The poor performance of the models on the Bizkaia test set can be attributed to differences in the characteristics of the training and test data. Notably, the mean value of the training data is significantly lower than that of the test data, with egg counts in 2023 being unusually high. This discrepancy between the training and test datasets likely contributed to the models’ suboptimal performance for Bizkaia. Nevertheless, for predicting egg abundance in the municipality of Bilbao (Bizkaia), SARIMAX demonstrated superior performance.

Finally, we applied the best-performing models to estimate *Aedes* invasive mosquito abundance in the Basque Country provinces for the upcoming year. By analyzing mosquito egg counts and environmental factors, this study improves and contributes the understanding of seasonal influences on mosquito abundance in a nonendemic region with a maritime climate, characterized by cooler temperatures, rainy weather, and the presence of competent mosquito vectors. These predictions could be used to inform public health strategies and mosquito control efforts, thereby helping to prevent the spread of mosquito-borne diseases in nonendemic regions.

These findings provide valuable insights for future research on assessing the risk of arboviral outbreaks in non-endemic regions such as the Basque Country. By considering factors such as imported cases, mosquito abundance, and seasonal variations, risk evaluations for mosquito-borne diseases can be further refined. Nevertheless, limitations remain in generalizing these results across the diverse areas within each province. For example, Bizkaia, which houses the largest human population in the Basque Country, includes regions with distinct microclimates that may affect invasive mosquito abundance differently.

Moreover, using shorter temporal intervals, such as weekly data collection (depending on vector monitoring schedules and data availability), could improve the precision of vector control strategies and strengthen the assessment of mosquito-borne disease risks. However, this would primarily depend on the intervals between vector population monitoring and the availability of data. Moreover, future improvements in this research should focus on a deeper analysis of methods for partitioning the dataset into training and testing sets, which may enhance the model’s performance.

This research aims to estimate mosquito population abundance and contribute to the development of vector control strategies, mitigating the risks of mosquito-borne infections, particularly given the region’s specific environmental conditions. Furthermore, the study emphasizes the critical need for ongoing, localized surveillance to better understand and address the expanding threat of mosquito-borne diseases.

## Conclusions

This study highlights the importance of environmental factors, particularly temperature, in predicting the abundance of invasive *Aedes* mosquitoes in the Basque Country. While statistical and machine learning models proved effective, forecasting remains challenging due to climate variations and data collection challenges. The findings underscore the necessity of continuous mosquito monitoring and improved predictive models to enhance public health strategies. Future research should focus on increasing surveillance frequency to strengthen risk assessments and vector control efforts in non-endemic regions.

## Supplementary Information

Supplementary Material 1.

## Data Availability

The environmental data used in this study were retrieved from several meteorological stations managed by Euskalmet, the Basque Agency of Meteorology. This data are openly available through the OpenData Euskadi platform [31]. The mosquito egg counts, collected using ovitraps, were provided by NEIKER, the Basque Institute for Agricultural Research and Development (see [7] for details). Due to ethical considerations and commercial sensitivity, these data are not publicly available.

## Footnotes

1 Note that not all meteorological stations displayed on the map contain records of all environmental features selected for this study.
