# Correction: Forecasting invasive mosquito abundance in the Basque Country, Spain using machine learning techniques

**DOI:** 10.1186/s13071-025-06770-7

**Published:** 2025-03-27

**Authors:** Vanessa Steindorf, Hamna Mariyam K. B., Nico Stollenwerk, Aitor Cevidanes, Jesús F. Barandika, Patricia Vazquez, Ana L. García-Pérez, Maíra Aguiar

**Affiliations:** 1 M3A, Basque Center for Applied Mathematics, Mazarredo 14, 48009 Bilbao, Bizkaia Spain; 2 Ikerbasque, Basque Foundation for Science, 48009 Bilbao, Bizkaia Spain; 3 Animal Health Department, NEIKER-Basque Institute for Agricultural Research and Development, Basque Research and Technology Alliance (BRTA), 48160 Derio, Bizkaia Spain

**Correction: Parasites & Vectors (2025) 18:109** 10.1186/s13071-025-06733-y

Following publication of the original article, it came to the journal's attention that the affiliations information of the author Aitor Cevidanes was incorrect: the author was (incorrectly) affiliated with affiliation 1 instead of affiliation 3 (their correct affiliation). The article [1] has since been updated to correct this. Thank you for reading this erratum.
