# Supplementary material for: Forecasting invasive mosquito abundance in the Basque Country, Spain using machine learning techniques
Source: Parasit Vectors. 2025 Mar 15;18:109. doi: 10.1186/s13071-025-06733-y (PMC11909879; doi:10.1186/s13071-025-06733-y)
Supplement: Supplementary file 1 — Supplementary Material 1. [file 13071_2025_6733_MOESM1_ESM.pdf]

Supplementary Material

S1 Models overview

In this study, we applied different models including and excluding the lagged version of eggs count as a proxy and the lagged version of the independent environmental variables. To appropriately handle the discrete and non-negative nature of counts, we restrict our choices and applications of the models presented here. For instance, the statistical methods such as the Poisson Regression and Negative Binomial Regression are foundational models for count data [37]. However, while the first one assume that the time series follows a Poisson distribution, the second one can be useful when time series presents more variability and over-dispersion (i.e., the variance is greater than the mean) (as it is the case). Both models are an extension of the Generalized Linear model (GLM) with a log link function.

The GLM is a flexible extension of ordinary linear regression that accommodates response variables with error distributions other than the normal distribution. This model often outperforms others when applied directly to the original data, compared to the transformed data such as using logarithmic scale [38, 39]. As such, we initially avoided any normalization or transformation of the data. When we applied the GLM to this dataset, it performed better on the smoothed data (using a three-point central moving average) than on the original, unprocessed data. And, a GLM with the canonical link function was used, assuming a Gaussian distribution for the response variable. In other words, the response variable follows a Gaussian exponential family distribution. This allows for more flexibility in modeling, as it does not impose the strict relationship between mean and variance required by models such as when using the Poisson distribution [40].

The GLM with a Gaussian family assumes a linear relationship between the predictors and the response variable $\mathbf{Y}$, using the identity link function. That is, the

conditional mean $\boldsymbol{\mu}$ is a linear combination of unknown parameters $\boldsymbol{\beta}$ via the link function g:

$$\mathrm{E}(\mathbf{Y} \mid \mathbf{X}) = \boldsymbol{\mu} = g^{-1}(\mathbf{X}\boldsymbol{\beta}) = \beta_0 + \beta_1 x_1 + \cdots + \beta_p x_p,$$

where $\mathrm{E}(\mathbf{Y} \mid \mathbf{X})$ is the expected value of $\mathbf{Y}$ conditional to $\mathbf{X}$, and g is the link function, which in this case, is the identity function [40]. The model predicts the mean of the response variable based on the input variables, and estimates the coefficients $\boldsymbol{\beta}$ by maximizing the likelihood function, assuming that the residuals are normally distributed.

Moreover, not only the dataset is over-dispersed but the response variables contains a lot of zeros, due to two main reasons: one the absence of positive eggs count outside during winter season (the so-called true negative) and the absence of more samples in more localities (the so-called false negative). In this case, zero-inflated models can handle excess zeros effectively. Zero-Inflated Negative Binomial (ZINB) can be effective in this case assuming that the data come from a mixture of two processes: one generating zeros and another generated by a negative binomial distribution [35]. Other models that can handle over-dispersion and the zero counts is the Generalized Additive Models for Location, Scale, and Shape (GAMLSS) which is flexible in modeling different distributions, not just the mean but also the variance [41]. However, after applying these models to the dataset, we observed that they were prone to over-fitting, indicating that the model might learned the noise in the training data rather than generalizing the unseen data.

On the other hand, our predictors are temporal series mostly exhibiting seasonal trends. Time series models like Seasonal Autoregressive Integrated Moving Average (SARIMA) are commonly used for forecasting since it is suitable for temporal count data and can handle seasonality. SARIMA has been extended to the Seasonal Autoregressive Integrated Moving Average with Exogenous variables (SARIMAX)

which can include exogenous variables giving more accurate outcomes. SARIMAX combines differencing, autoregression, moving averages, and seasonal components, incorporating as well exogenous predictors [42]. Unlike models such as the GLM, this model assumes that the response variable depends on its past values Y_t. Also, of its past forecast errors ϵ_t, and external predictors, capturing temporal effects. This relation reads:

$$Y_t = \phi_1 Y_{t-1} + \cdots + \phi_p Y_{t-p} + \theta_1 \epsilon_{t-1} + \cdots + \theta_q \epsilon_{t-q} + \boldsymbol{X\beta} + \epsilon_t,$$

where Y_t is the response variable at time t, ϕ_i are the the autoregressive (AR) parameters, θ_i the moving average (MA) parameters, $\boldsymbol{X}$ is the predictors (exogenous variables), and $\boldsymbol{\beta}$ is the vector of coefficients.

The model depend, as well, on the order of the AR terms p, representing the number of lagged values of the series used in the model; the degree of differencing d, which removes trends and makes the series stationary; the order of the MA terms q, representing the number of lagged forecast errors. And, on the seasonal component, P, D, and Q that are the seasonal terms for the parameters p, d, and q, respectively, and on is the length of the seasonal cycle s [42]. We implemented SARIMAX in the R compute language by using the `auto.arima( )` function that automatically selects the best seasonal and non-seasonal parameters p, d, q, P, D, Q, and s based on the data.

On the other side, machine learning techniques such as Random Forest (RF) [43], Conditional Inference Trees (CTree), and Artificial Neural Networks (ANNs) can be also used for forecasting count data. However, in this study, ANNs is the least performing machine learning model, a finding corroborated by previous research [35] which do not advises using ANNs for count data with over-dispersion.

RF builds decision trees using bootstrap samples and random feature subsets, and combines the predictions from all trees. Each tree is developed using a subset of features, as chosen by the `mtry` parameter [43]. The `mtry` parameter determines the

number of predictor variables considered at each split, playing a crucial role in controlling over-fitting. The `ntree` parameter refers to the number of trees to be generated in the RF. Increasing the number of trees generally enhances model stability and robustness, although beyond a certain threshold number of trees, the additional trees yield to insignificant improvements in the terms of model performance. The advantage of RF lies in its ability to handle complex data and it is designed to mitigate over-fitting [17, 35].

The final RF predictions for the conditional mean of $\boldsymbol{Y}$, given the predictor $\boldsymbol{X}$, is based on the average or weighted average of all the individual trees' predictions. Thus, the RF model can be expressed as:

$$\hat{\mathrm{E}}(\mathbf{Y} \mid \mathbf{X}) = \frac{1}{K} \sum_{k=1}^{K} \omega_k h_k(\boldsymbol{X})$$

where $h_k(\boldsymbol{X})$ is the prediction of the k-th tree, and K is the total number of trees [43]. Each tree is built using a bootstrap sample of the original data and selects features at random from the `mtry` subset.

On the other hand, the CTree is a non-linear method to model the relationships between predictor variables and a response variable. The CTree algorithm recursively partition the dataset based on the values of the predictors, using statistical tests to determine the significance of potential splits. The splits are chosen by testing the association between each predictor and the response, and the predictor with the strongest association (lowest P-value) is selected for each split.

The conditional distribution of $\boldsymbol{Y}$, given the predictor $\boldsymbol{X}$, is estimated as:

$$\hat{\mathrm{E}}(\mathbf{Y} \mid \mathbf{X}) = \sum_{j=1}^{J} \hat{Y}_j w_j(\boldsymbol{X})$$

where $\hat{Y}_j$ is the predicted value for the j-th terminal node, $w_j(\boldsymbol{X})$ is the weight indicating whether observation j falls into the same terminal node as $\boldsymbol{X}$ [44].

While RF and CTree both rely on decision tree methodologies, they differ in their approaches. RF employs random feature selection to create an ensemble of trees, which enhances generalization but sacrifices interpretability. Conversely, CTree focuses on unbiased variable selection, offering better interpretability. RF generally offers better predictive performance on large and complex datasets, while the structural differences in the partitions can highlight the unique advantage of CTree.

S2 Dataset summary per province

S2.1 Climatic variables

Figure S1 shows the distribution of climatic data, including temperature, humidity, and precipitation, across the provinces of Gipuzkoa, Bizkaia, and Araba. The graphs summarize the weather variables, highlighting outliers (represented as single points or circular dots) in the dataset. The horizontal line dividing the box in two represents the median value of the time series for each climatic variable.

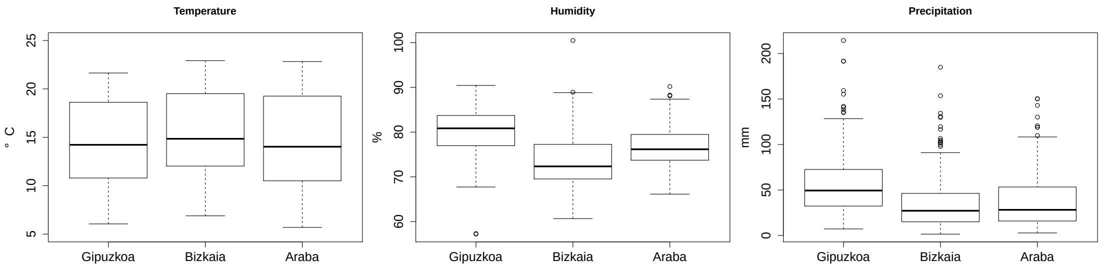

Fig. S1: Distribution of time series values for average temperature (°C), relative air humidity (%), and cumulative precipitation (mm) over an interval of 14 days, for all three provinces of the Basque Country.

S2.2 Lagged time series for Gipuzkoa

Figure S2 (a) shows the monotonic correlation, using Spearman correlation, between the climatic variables and the egg count time series for Gipuzkoa. Figure S2 (b) highlights the time lag at which the highest correlation occurs. At this time lag, the lagged time series will be used as predictors.

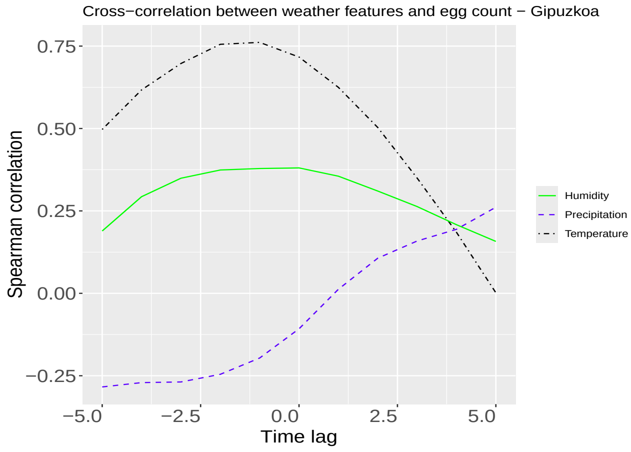

(a)

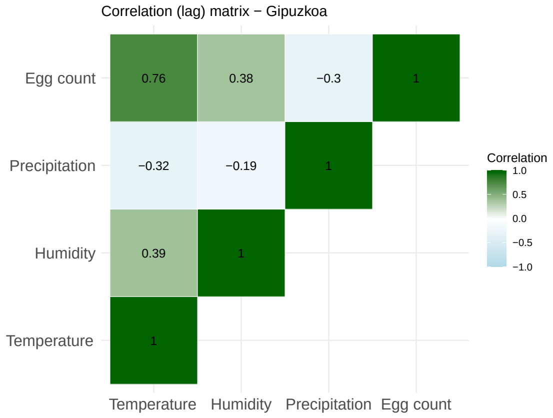

(b)

Fig. S2: (a) Spearman correlation indices for the time lag between temperature, humidity, precipitation, and the number of eggs. (b) The highest Spearman correlation values between the lagged time series.

S2.3 Lagged time series for Bizkaia

Figure S3 (a) shows the monotonic correlation using Spearman correlation between the climatic variables and the egg count time series for Gipuzkoa. Figure S3 (b) highlights the time lag at which the highest correlation value occurs. The lagged time series corresponding to this highest correlation will be used as predictor variables.

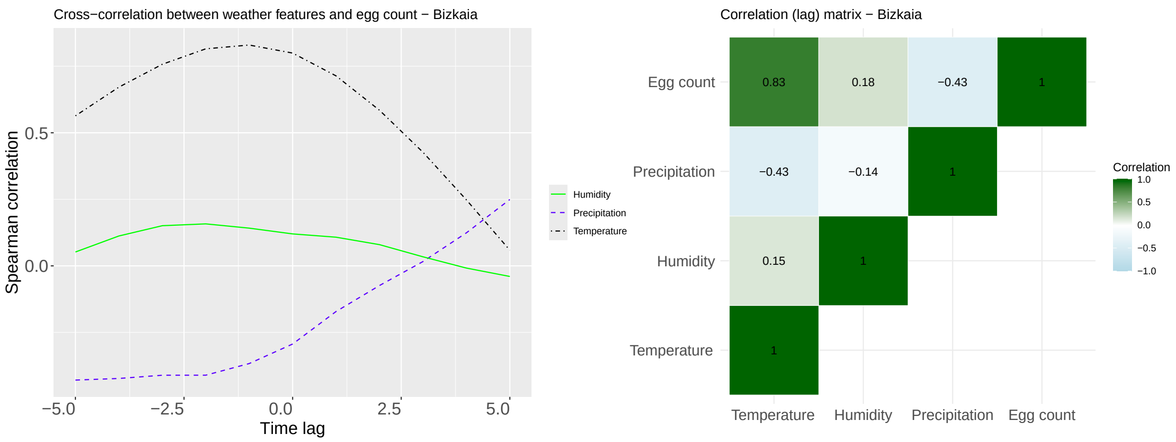

(a) (b)

Fig. S3: (a) Spearman correlation indices for time lags between temperature, humidity, and precipitation with the number of eggs. (b) The highest Spearman correlation values for the lagged time series.

S3 Data selection per municipalities

Due to the dispersed data with many zero values for egg counts and the lack of sufficient information to create a reliable training dataset at the municipality level, we conducted the analysis at the provincial level. However, we selected one municipality from each province to present the results.

For Gipuzkoa, we selected Irun, a municipality of interest due to its proximity to the French border and the frequent movement of travelers. Irun also had more positive ovitraps during the analysis period compared to the capital, Donostia/San Sebastián. The C084 weather station in Irun was selected over C083, as the latter's dataset lacked temperature, precipitation, and humidity data.

For Bizkaia, we chose Bilbao as the municipality, rather than larger municipalities like Barakaldo or Basauri, since both lack meteorological stations within their boundaries. For Bilbao, station C0B0 had no data on precipitation or temperature, while station C039, located in Deusto, provided data from 2016 to

2021, and station C03A began recording data in December 2021. To cover the entire study period, data from both C039 and C03A were used in the initial analysis.

For Araba, we chose the municipality of Laudio/Llodio. The primary reason for selecting this municipality is that no egg counts were recorded in the ovitraps in the capital, Vitoria. For weather data, two meteorological stations in Laudio/Llodio were listed in the database (see more details in [31]): station C067, which was selected, and station C027, which was not included due to missing data for the chosen period.

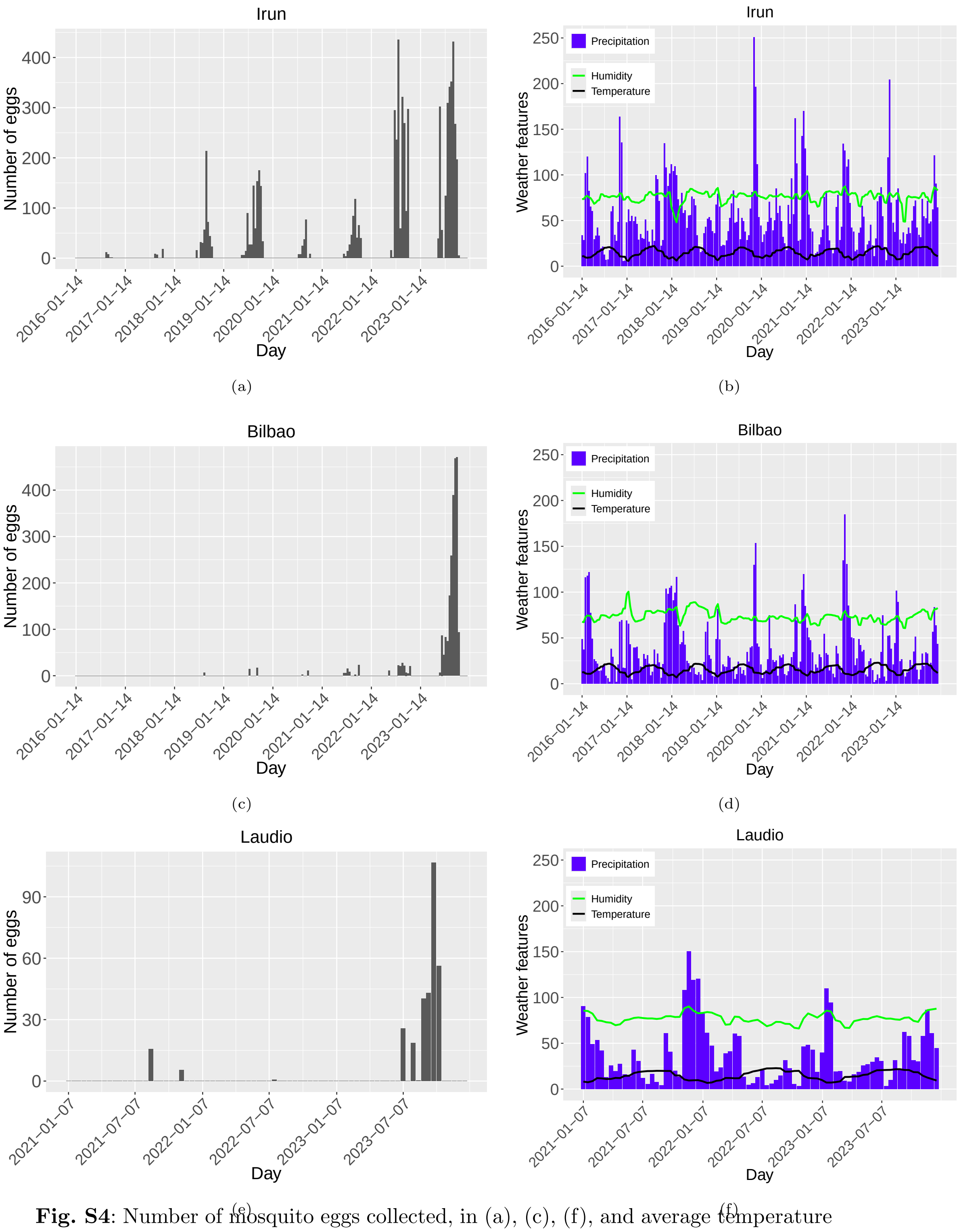

Fig. S4: Number of mosquito eggs collected, in (a), (c), (f), and average temperature ($^{\circ}C$), relative air humidity (%), and cumulative precipitation (mm), in (b), (d), (f). For Irun (in Gipuzkoa), Bilbao (in Bizkaia) and Lodio (in Araba).

Egg count data were collected at the municipality level, disregarding specific ovitrap locations. The dataset was constructed by averaging the highest three egg counts from the ovitraps for each municipality every 14 days (bi-weekly). This approach was necessary because the number of monitored ovitraps varied throughout the study period. We selected the top three counts since, on average, no more than 10 ovitraps were placed in each municipality every 14 days. Weather data were aggregated by municipality on a daily basis. A dataset was then constructed containing the average temperature, average humidity, and cumulative precipitation for the previous 14 days.

This information was combined into a single dataset for each municipality. The time series of egg counts, average temperature, humidity, and cumulative precipitation are presented in Figures S4(a)-(b), S4(c)-(d), and S4(e)-(f).

For further analysis, we will focus on the municipalities of Irun and Bilbao, since Laudio has only recorded positive ovitraps from 2021 onward. The same methodology and analysis applied at the provincial scale will now be carried out at the municipality scale.

S3.1 Irun

Statistical analysis

For Irun, Figure S5 shows the relationship between meteorological variables and number of mosquito eggs count using scatter plots. While Figure S6 shows the monotonic correlation using Spearman correlation between eggs counts and the time lagged versions of the climate features. For temperature, maximum correlation occurs at -1 units (2 weeks). For humidity, maximum occurs at -2 units. And for precipitation, maximum correlation occurs at -5 units, with negative correlation.

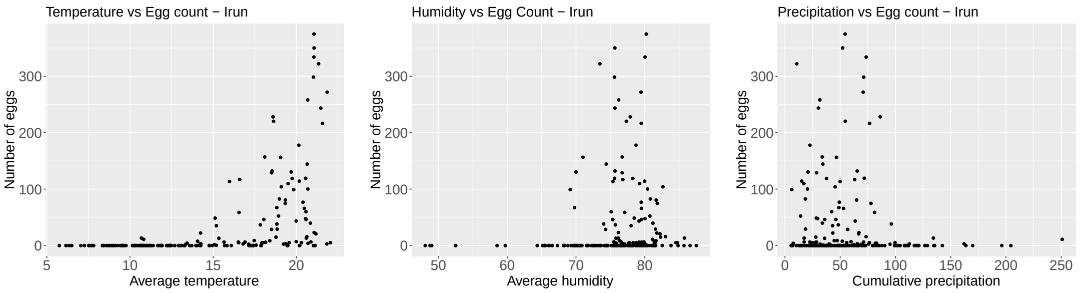

(a) (b) (c)

Fig. S5: (a) Average temperature (in $^{\circ}C$) versus number of collected mosquitoes eggs. As temperature increases, the number of eggs increases. (b) Average air relative humidity (in %) versus number of collected mosquitoes eggs. As humidity increases, the number of eggs increases. (c) Accumulated precipitation (in mm) versus number of collected mosquitoes eggs. As precipitation increases, the number of eggs decreases.

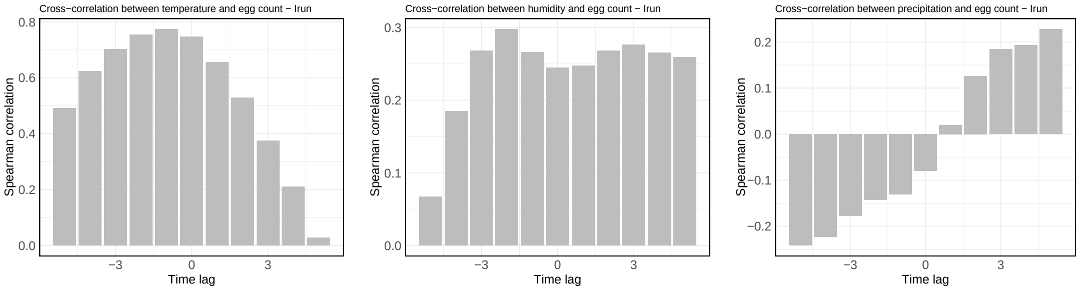

(a) (b) (c)

Fig. S6: Spearman correlation between the weather feature time series and the number of mosquito eggs, with a time lag of 1 unit (2 weeks). (a) For temperature, the maximum correlation occurs at a lag of −1 unit. (b) For humidity, the maximum correlation occurs at a lag of −2 units. (c) For precipitation, the maximum negative correlation occurs at a lag of −5 units.

Fitting

We implemented the GLMG, SARIMAX, RF, and CT models using the R programming language for the Irun dataset. The training dataset spans from 2017 to 2022, while the test dataset consists of data from 2023, as shown in Figure S7. We compared the models' performance on both the training and testing datasets, evaluating them using the Mean Absolute Error (MAE), Root Mean Squared Error (RMSE), and R-squared score (R^2), as detailed in Table S1.

Table S1: Error metrics for each chosen model, on the training and test datasets for Irun.

Model	MAE Train	MAE Test	RMSE Train	RMSE Test	R^2 Train	R^2 Test
RF	4.00	43.27	10.22	68.84	0.97	0.70
SARIMAX	8.45	37.55	17.32	57.35	0.91	0.79
GLM	10.10	33.51	21.57	52.87	0.86	0.82
CT	11.77	54.21	25.86	89.16	0.80	0.49

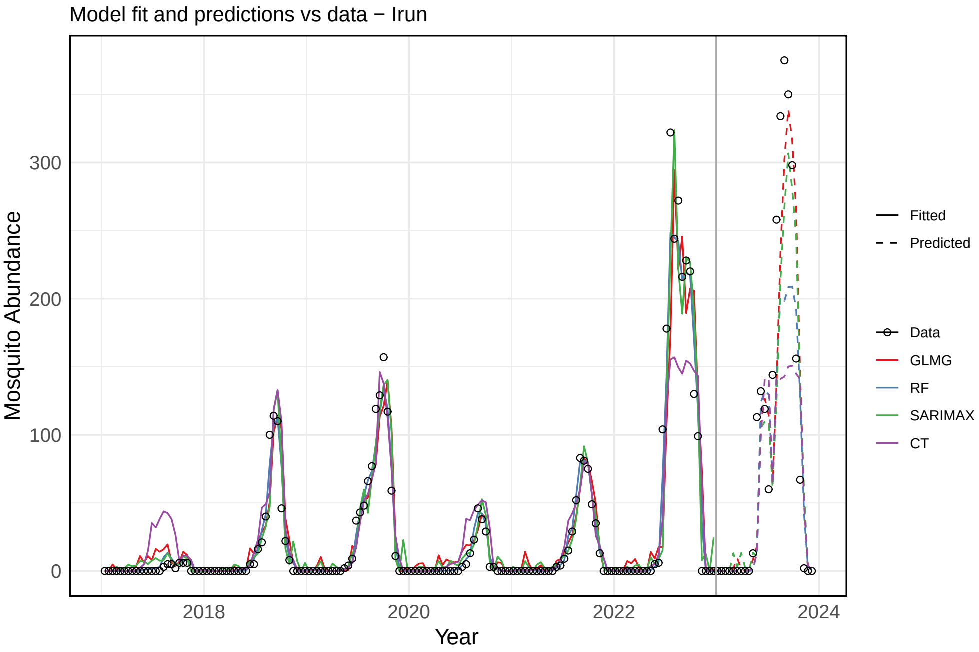

Fig. S7: Comparison of actual data versus fitted and predicted values for the Irun dataset. The actual data is represented by open black circles, while the fitted values for each model are shown as solid lines and the predicted (test) values as dashed lines. The models are colored as follows: RF model (`ntree` = 600, `mtry`= 5) in blue, GLMG model in red, SARIMAX model in green, and CT model (`ntree` = 500, `mtry` = 3) in purple. The vertical gray line separates the training dataset (2017–2022) from the testing dataset (2023).

Forecasting

Subsequently, we used the best-performing trained models to forecast future *Aedes* invasive mosquito abundance in Irun. To do this, we included 2023 data points as part of the training dataset. Using the historical time series data and their lagged versions, we predicted future values based on the most recent observations (see Figure S8). The error analysis for the training dataset is presented in Table S2, which shows that the Random Forest (RF) model performed the best, explaining 97% of the variance in the training dataset.

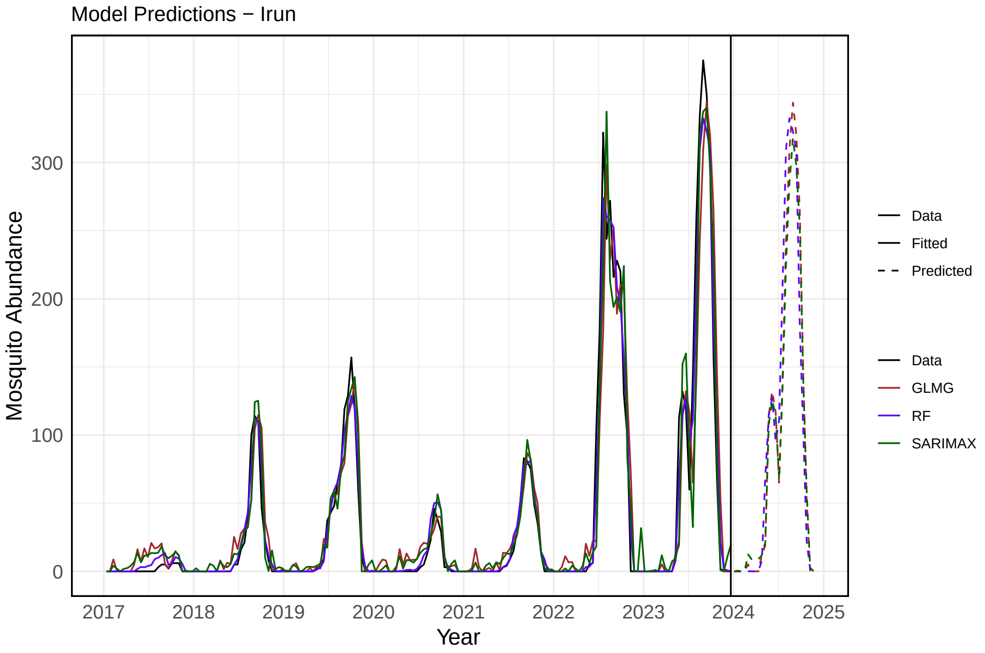

Fig. S8: The actual data versus the fitted and predicted values of the model for Irun. The data is represented by a solid black line, with fitted values shown as solid lines in color and predicted values as dashed lines. In blue, the RF model (`ntree` = 600, `mtry` = 5); in brown, the GLMG model; and in green, the SARIMAX model. The vertical black line separates the training dataset (from 2017 to 2023) from the forecasted period for the year 2024.

Table S2: Error metrics in the training dataset, for Irun).

Model	MAE	RMSE	R^2
RF	5.90	12.17	0.97
SARIMAX	11.89	23.56	0.90
GLM	13.68	27.71	0.86

S3.2 Bilbao

Statistical Analysis

For Bilbao, Figure S9 presents the relationship between meteorological variables and mosquito egg counts using scatter plots. Figure S10 illustrates the monotonic correlation, calculated using Spearman's correlation, between egg counts and the time-lagged versions of the climate features.

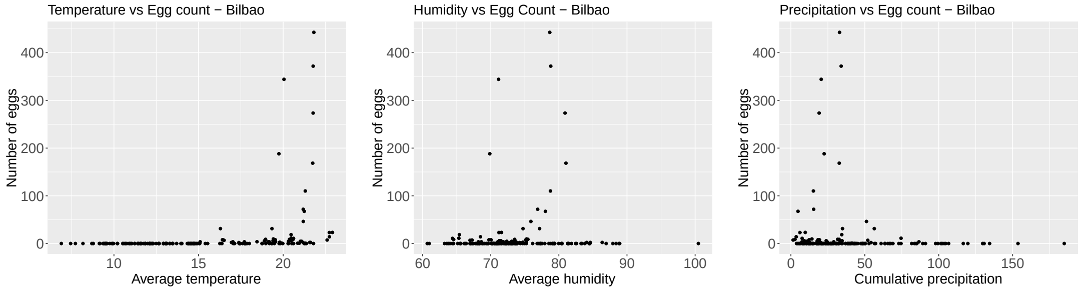

(a) (b) (c)

Fig. S9: (a) Average temperature (in $^\circ C$) versus number of collected mosquito eggs. As temperature increases, the number of eggs increases. (b) Average relative humidity (in %) versus number of collected mosquito eggs. As humidity increases, the number of eggs increases. (c) Accumulated precipitation (in mm) versus number of collected mosquito eggs. As precipitation increases, the number of eggs decreases.

Fitting

We also implemented the GLMG, SARIMAX, RF, and CT models for the Bilbao dataset. The training dataset consists of data from the year 2019 to 2022, while the test dataset consists of data points from 2023, as shown in Figure S11. We compare

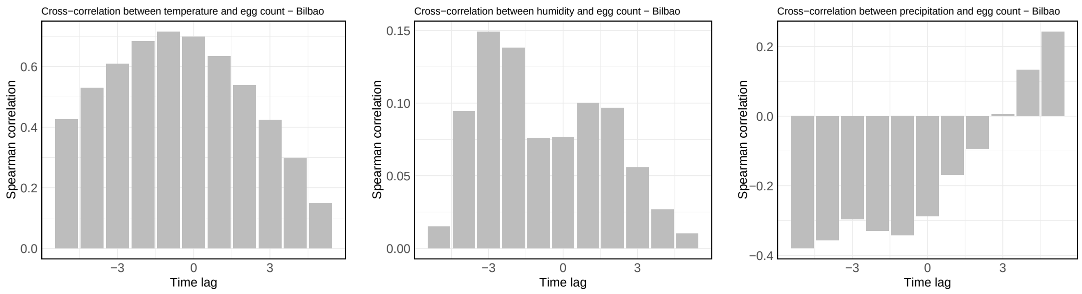

(a) (b) (c)

Fig. S10: Spearman correlation between the weather features time series and number of mosquito eggs with a time lag of 1 unit (2 weeks). For (a) temperature, maximum correlation occurs at -1 units. For (b) humidity, maximum correlation occurs at -3 units. For (c) precipitation, maximum correlation occurs at -5 units.

the models on both the training and testing datasets, evaluating each model's performance using the MAE, RMSE, and R^2 metrics, as detailed in Table S3.

Table S3: Error metrics in the train and in the test dataset, for Bilbao.

Model	MAE Train	MAE Test	RMSE Train	RMSE Test	R^2 Train	R^2 Test
RF	0.57	81.38	0.99	150.86	0.95	-0.32
SARIMAX	1.10	45.02	1.75	81.82	0.86	0.61
GLM	1.25	36.78	2.25	64.42	0.76	0.76
CT	1.41	82.00	2.72	152.01	0.65	-0.34

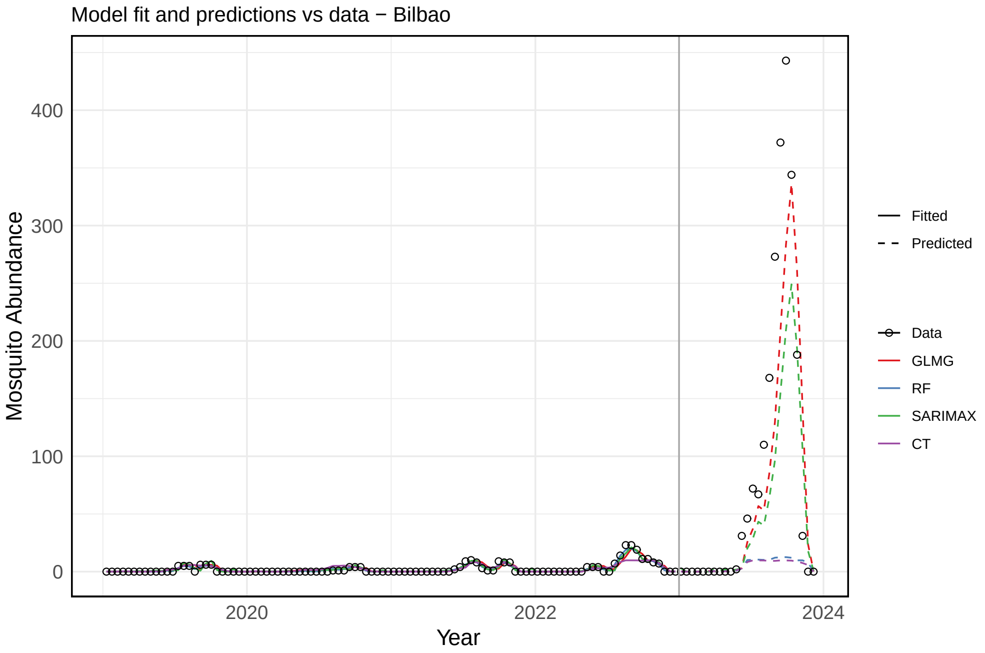

Fig. S11: The actual data versus the fitted and tested values of the model for Irun. The data is represented by open black circles, while the fitted values of each model are shown in solid lines and the predicted (tested) values in dashed lines. In blue, the RF model (`ntree`= 600, `mtry` = 5); in red, the GLMG model; in green, the SARIMAX model; and in purple, the CT model (`ntree` = 500, `mtry` = 3). The vertical gray line delineates the training dataset (from 2017 to 2022) from the testing dataset (2023).

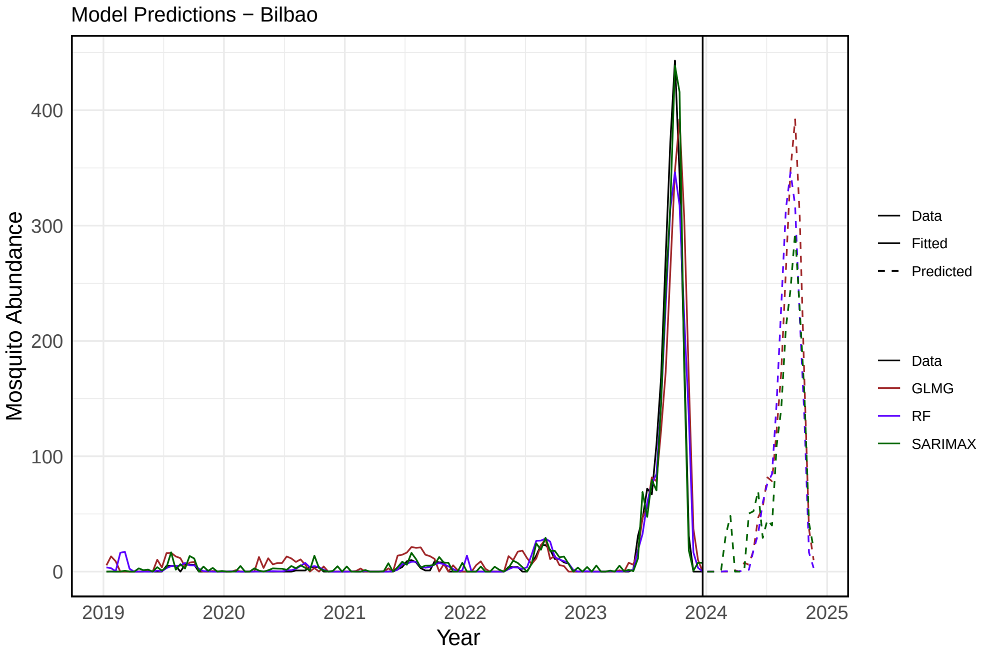

Fig. S12: Actual data versus fitted and predicted values for Bilbao. The actual data is represented by a solid black line. The fitted values for each model are shown in solid colored lines, while predicted values are in dashed lines. The RF model is shown in blue (`ntree` = 600, `mtry` = 5), the GLMG model in brown, and the SARIMAX model in green. The vertical black line separates the training dataset (from 2017 to 2023) from the forecasted period for the year 2024.

Forecasting

The best-trained model was then used to predict future *Aedes* invasive mosquito abundance in Bilbao using the historical time series data and lagged versions, as shown in Figure S12. The error analysis for the training dataset is presented in Table S4, indicating that the model with the best performance is the SARIMAX model, which explains 97% of the variability in the training dataset.

Table S4: Error metrics for the training dataset, for Bilbao.

Model	MAE	RMSE	R^2
RF	4.98	15.28	0.95
SARIMAX	4.63	10.57	0.97
GLM	9.45	24.23	0.87
